# Short History of Epidemiology for Noninfectious Diseases in Japan. Part 1: Selected Diseases and Related Episodes from 1880 through 1944

**DOI:** 10.2188/jea.17.1

**Published:** 2006-12-29

**Authors:** Kunio Aoki

**Affiliations:** 1 Professor Emeritus, Nagoya University, and Honorary President, Aichi Cancer Center.; 2 Chairman, The Nagoya City Council of Social Welfare.

Publication in Japan of works on epidemiology/preventive medicine is on the record by 1810 e.g. "Taisei Ekiron (Western Epidemiology)" by Ryotei Shingu, and "Dandokuron (Drawing out the Poison)" by Hakuju Hashimoto. Following a European tour of inspection, Sensai Nagayo came to attach importance to health and hygiene. After setting up a Medical Affairs Office at the Ministry of Education, Nagayo transferred the office to the Ministry of Interior as the Bureau of Hygiene in 1873. Three departments under the bureau were in charge of medicine, hygiene, and health statistics, respectively. At the time of a cholera epidemic, the office issued preventive directions and passage of the Quarantine Law in 1875. Promulgation of a Cholera Preventive Ordinance took place in 1880. In the same year, Shokei Shibata published "Introduction to Hygiene", which discussed how health and disease related to lifestyle, occupation, or physical constitution. The government also began to publish statistics of hygiene: data on infectious diseases.

No exact date is known for when the concept of epidemiology itself was introduced into Japan. In London, a Society of Epidemiology was established in 1850. In Germany, Haeser published: "Historisch-pathologischen Untersuchungen. Als Beitraege zur Geschichite der Volkskrankheiten. 2 Bdr Dresden und Leipzig, 1839-41". Certain Japanese medical students who had studied abroad in the course of the late 19th century brought back with them the epidemiology concept, together with that of hygiene. They put to use the idea immediately, in researches that sought out the causal factors behind diseases. At the same time, the concept of geographic pathology also came to be brought to Japan and used as a method in pursuit of the environmental factors responsible for the outbreak of epidemics of diseases with unknown etiology.

Rintaro (Ohgai) Mori, a military physician, in 1889 translated the German word "Epidenmilogie" into the Japanese language as "Ekireigaku". Yu Fujikawa translated the same term as "Ekibyogaku" or "Ryukobyogaku". Another translation in use was "Ekirigaku". Hirsch, author of "Handbuch der Historisch-geographischen Pathologie" published in Erlangen, Germany as of 1859, defined epidemiology as a study of the nature of diseases via multiple approaches, namely medical geography, geographic pathology, and geographic anthropology. Fujikawa explained epidemiology as having two facets: descriptive and analytic epidemiology. The latter analyzes causal factors employing clinical, pathological, and microbiological methods.

In those days, epidemiologic methodology was applied to those acute infectious diseases that repeatedly triggered epidemics with numerous fatalities. The methodology was also frequently applied to the study of noninfectious diseases for which no causes were known. This article is an overview of the progress in Japan of epidemiologic research, chiefly on noninfectious diseases, which began during late 19th century. At the same time, the status of epidemiology in Japan is also discussed.^1^^,^^6^

## CLINICO-PATHOLOGICAL AND GEO-STATISTICAL RESEARCH ON NONINFECTIOUS DISEASES

During the period from the late 19th century to early 20th century, when modern medicine in Japan was still in its infancy, researchers were already actively engaged in etiological studies not only on infectious diseases, but also endemic diseases of unknown cause, including environmental factors. The methods then employed began with collection of cases. From data thus collected, clinical and pathological images of the disease emerged, which, in turn, enabled researchers to form a concept of the disease. The next step was to survey the illness' frequency distribution together with other epidemiologic characteristics. Each patient's specific physiological, pathological and microbiological features were recorded in order to gain a comprehensive picture of the disease. Attempts were made to surmise etiology, e.g. factors responsible for an outbreak. Preventive methods against the disease were drawn from data consolidated from the records.

This article will introduce studies that the author believes are representative of this era, including those related to epidemic episodes. The research described is limited to works for which the source materials were available to the author.

### A. Research on Beriberi

The bibliography at the end of Section A includes the following valuable records on beriberi: Seizo Yamashita's extensive works; Kanehiro Takaki's collected works dealing with the relation between beriberi and epidemiology and its prevention; Itokawa's summary; Shun'ichi Yamamoto's articles on Rintaro Mori. This article might appear superfluous in that the beriberi problem has been oft discussed. Because beriberi was an important topic in our own history of epidemiologic research, however, the author ventures to look back on beriberi-related disputes and to examine their historical significance.

Beriberi is a disease long known to have been present in Japan, and from around the mid- 17th century, cases of beriberi began to increase markedly in this country. Prevalence fluctuated, continuing into the Meiji Era (1868-1912). The cause of beriberi is now known to be unbalanced diet, with heavy reliance on polished rice as staple food. During the Edo Era (1603-1868), a diet of polished rice spread from the courtier class, wealthy persons, or high rank samurai to lower ranking Samurai and townspeople. These people consumed a great quantity of rice, with not too many side dishes. While beriberi first gained force in Kyoto, major cities such as Edo (present day Tokyo) or Osaka came to experience high incidence by the latter half of the 17th century. Among the rural population and farmers, however, beriberi was rare: owing to their poverty, peasants could not afford rice as staple food. A policy of discouraging rice as staple food also played a role. Because of the prosperity brought about by the industrializing Meiji Era, polished rice gradually became generally available as incomes, rice production, and imports grew. According to Kanehiro Takaki, what enabled farmers to eat rice was the land tax reform of 1873 which allowed cash payment of the tax in place of rice even in rural areas; as a consequence farmers were able to store surplus rice. Thus, a popularization of the polished rice diet contributed to a nationwide spread of beriberi. Both the Japanese Army and Navy adopted the polished rice diet. As popular as was polished rice, it was at the time still beyond the reach of some farmers and common folks. Thus, it was attractive to young soldiers. With 6-*go* (1 *go =* 180mL) daily allotment of rice per soldier, the beriberi prevalence among drafted soldiers grew unexpectedly high, 10-20%. A high prevalence of 50% was found among naval personnel out to sea for long periods. With a high recurrence rate of beriberi and practically nonexistent treatment for it, patients had to be suspended from work to rest. The disease was allowed to take its natural course. This situation resulted in no small number of deaths. Beriberi became a serious problem for national defense. Beriberi was hardly known in Europe. Therefore, Japanese students studying medicine in Europe did not acquire any knowledge of beriberi. Once returned to Japan they had to cope with it entirely on their own. The time being one of breakthroughs in infectious disease research, the majority of researchers assumed beriberi to be an infectious origin. They concentrated on finding a pathogen. While some etiological theories other than infection theory were proposed, e.g. rice poisoning or nutritional disturbance, it was hard to get close to the disease's true cause. In this environment, through clinical and epidemiologic observations, it was the Navy that reached the conclusion that the roots of beriberi lay in diet. The Navy managed to lower beriberi incidence close to zero by improving its ranks' diet. However, many disputes regarding the Navy's research hypothesis and outcome data would still ensue, as recounted below. Their final resolution would only come some 40 years later.

#### a. Navy Beriberi Research and Proposed Countermeasures

According to Navy's morbidity records kept since 1878, the rate of beriberi prevalence over the first six years after the beginning of record-keeping was 22-40%. The disease could recur in the same person during the same year. Cumulative mortality was 1.57-3.96%. Detailed statistics became available after the improvement of records after 1883. Out of 5,346 persons working in naval departments and agencies, the total number of days in hospital due to beriberi was 21,273, an average of 4 days per person per year. Naval hospitals could not house these patients and had to use available space at medical wards in nearby temples or other buildings. During Kanehiro Takaki's tenure at naval hospitals, 1872-1876, he was kept pressed for time to treat the already numerous beriberi patients.

Takaki went to the United Kingdom to study at St. Thomas Hospital School in 1875 and returned to Japan in 1880 after graduated from the school with an excellent record. Soon he was to head the Tokyo Naval Hospital. As a clinician as well as an administrator, Takaki fully realized the importance of the beriberi problem, and became actively engaged with it.

Firstly for two years he surveyed and tallied beriberi incidence rates among 4,683 naval personnel in relation to aboard 18 naval ships, headquarters, barracks, students, and prisoners. Striking differences in incidence existed, depending on workplace in various departments or agencies. However, no definite tendency was noted. Class-wise, the rate was always high among sailors, prisoners, and students, while it was low among officers. No discernible relationship was found regarding season, living quarters, clothing or working condition, thereby excluding the possibility of person-to-person infection. The only difference between sailors/students and officers was content of meals served. Food for sailors/students was inexpensive containing little protein compared to more expensive and protein rich food for officers. Upon checking old records on life at sea, beriberi onset generally was less during the early part of a voyage, and then increased during the latter part of the voyage keeping pace when food supply began to diminish. Incidence suddenly improved while anchored in harbor or during landing excursions. Diet while in harbor or during landing excursions differed from that during a cruise.

From these observations, Takaki suspected the cause of beriberi to be a kind of food consumed. Though an amateur in the field, he began analytical study of food. At a time when food was not extensively studied, Takaki analyzed military rations according to nutritional elements, based on Edmund A. Parks' "Nutritional Treatise (translated into Japanese by Yoshinao Kobayashi in 1886)". As a scholar in the field of hygiene, Parks had aided Florence Nightingale in the Crimean War. The appropriate ratio of protein (N) versus Carbohydrates (C) in British military rations was supposed to be 1:15. On the other hand, the diet for sailors or prisoners in Japan contained extremely little protein but rather high carbohydrates, the ratio of N versus C approximately being 1:25. No beriberi case was found when the ratio improved to 1:22-23. Based on his findings, Takaki attributed a great part of beriberi symptoms to excessive carbohydrates and paucity of protein in the naval diet. While the pathogenesis of beriberi was unknown, Takaki planned to prevent beriberi by improving nutrients in the imbalanced military diet, namely the N/C ratio. He decided to increase protein by serving more meat or fish, and carbohydrate reduction by serving less rice and substituting for rice bread or biscuits as in England thereby changing the traditional diet into a Western style one. Beriberi-free England could have been in Takaki's mind as he made this decision.

However, his plan of changing Japanese military rations into English style was a big problem and met much opposition from those involved in this project. By 1882, however, his explanations of the relationship between the quality of food and beriberi incidence, as well as the effectiveness of his idea for beriberi prevention, gradually won people to his plan. However, the issue of whether sailors would accept Western style food had to be put to the test. Taking into consideration the N/C ratio, Takaki prepared several menus blending Japanese and Western style food for comparison to the traditional one. Over two weeks, five beriberi patients took traditional food, while the other five Western style food. Fortunately the test patients could tolerate the Western style diet. Patients in the Western style diet group underwent satisfactory improvement, with no deaths. Patients in the Japanese diet group were discharged from the hospital, but one died. No health problem was found in the Western food group, except for a slight weight loss. Takaki, now promoted to be Director of the Naval Medical Bureau, proposed to make larger scale improvements in meal plans. He obtained permission to execute them over a part of the Navy.

An experimental project began, involving personnel aboard two naval vessels in November 1883, when rules for food issuance, types of food to be purchased as well as storage were readied. Health education regarding the importance of food in preventing beriberi also began at the same time. Around the same time, Takaki set up an investigative committee aboard Ryujo — a training ship — to determine the cause of a beriberi epidemic there. The ship set out on a long cruise of nine months in 1883. Beriberi incidence during the cruise numbered 169 out of 376 crew members with 25 deaths. All the deceased were either petty officers or sailors. The committee found no difference between beriberi patients and the healthy as concerned environmental factors, such as living conditions or workplace. However, one factor was obviously different: food. The N/C ratio for sailors was 1:28, for students also with a high beriberi ratio was 1:25, while that for officers was 1:20. Takaki's expectations were confirmed. He thus believed in the efficacy of improved rations, based on his own observations of gradually decreasing beriberi incidence aboard the two naval ships. Therefore, he felt a need to test whether the effect would be the same over a long distance cruise as well. He submitted an experimental design for preventive medicine and lobbied for its implementation aboard the next vessel scheduled for long cruise, the training ship Tsukuba. There were various obstacles to the execution of the experiment, in particular its substantial cost (approximately ¥50,000). However, passionate persuasion succeeded in obtaining permission to go ahead with the project. Advance payment was secured by moving funds forward from the next year's budget.

The experimental design for the Tsukuba called for repeated cruises during the same season, the same route, and a staff of the same number as had the training ship Ryujo, which had seen a high beriberi incidence. The only difference was an improved Western style diet. Somewhat belatedly, Takaki also began to examine the influence of improved diet on animal subjects. When Tsukuba set sail in 1884, many persons involved in the experiment still worried about the outcome of a human experiment. Takaki also appeared to have braced himself to take responsibility in case the experiment turned out a failure. Despite all these worries, nearly every member came back healthy, with almost no outbreak of beriberi during voyages to Australia, South America, and Hawaii. The first Japanese human experiment on diet intervention was a success. The conclusion was that beriberi was preventable. Because improved naval rations were put into practice, by the next year beriberi incidence over the whole Navy decreased drastically, by 1885 reaching near zero. Desperate efforts on the part of persons in charge aboard ships were needed to persuade sailors to eat improved but unfamiliar food. Some sailors hated the bread and threw it into the sea. Discarded bread at times floated alongside the ship, necessitating Takaki to devise yet another improvement in naval diet. At around the same time, the Army in Osaka, troubled by high beriberi incidence, noticed the low number of beriberi patients among prisonersThe survey found that the incidence number decreased if their diet was changed to a mixture of barley with rice. Beriberi among common soldiers stationed in Osaka also decreased when their diet was changed to mixed barley and rice. Upon learning of the army result, Takaki's preventive measure also adopted the rice and barley diet beginning in 1885. Hardly any beriberi case was reported in the Navy after the switch. Table 1 shows an outline list of Takaki's research in the manner of present day research design.

**Table 1.** Takaki's survey methods for the causal agent of beriberi. (arranged by the author)

1. Observational epidemiology survey	
(a)	The survey population was the entire Navy. Observations were taken at 8 land stationed departments and bureaus and aboard 18 naval ships.
(b)	For departments and bureaus, observations of beriberi incidence were taken by month, season, and year beginning with 1878: For ship-stationed crew, data were extracted from past records of beriberi incidences while at sea, in harbor, or during land excursions.
(c)	For departments and bureaus as well as for ships, comparisons were made of environmental factors (weather, atmospheric temperature, humidity, etc.), biological environment, hygienic environment (workplace, living quarter, clothing, sanitary level, etc.), lifestyle (diet, drinking, hobbies, individual preferences) as well as that of working conditions
(d)	Examinations were made of individual cases for common causal factors
2. Analytical epidemiology survey	
(a)	Comparison of environmental factors for two groups (upper echelon officers versus prisoners, sailors, and students) with different incidence rates: possibility of man-to-man infection; analysis of nutrients in diet
(b)	Hypothesis formulation
(c)	Preliminary diet intervention study: new eating situation of patients or prisoners; therapeutic effects; animal experiments
(d)	Cooperative survey of causative factor for multiple beriberi incidences aboard Ryujo; discussions; relevance of diet
(e)	Verification of the effects of improved diet aboard Tsukuba in the diet intervention study
(f)	Reinforced hygiene instruction in the Navy; further improvements in naval rations; prevention of beriberi by barley-mixed diet

Looking through Table 1 item by item, Takaki's study gives an impression of being a series of scientific research studies based on a well ordered scheme. In reality, however, he left only sketchy survey methods, simple statistical tables and an explanatory summary. Therefore, evaluation of the quality of his work cannot be made due to lack of details of the analytical methods employed in his research. The following is a quote from Seizo Yamashita's book's critical remarks on Takaki's study made in the form of annotations on it. "The description of Takaki's work is based mostly on his post-retirement reminiscences, after the author had accomplished his goals and made his fame. As such, they give a feeling of having somewhat embellished reality. Take his survey of environmental factors. A vast number of researchers and much time are needed to do firsthand investigations of the entire Navy, not merely the stated one year and a half. My search failed to locate any such documents, whether results of epidemiologic surveys or raw records. Even if he did carry out documentary surveys, the Navy would at the time hardly have kept useful ones. Therefore, any such useful data would have come from a later survey. Takaki has presented only a part of the necessary supporting evidence, in particular the results of the epidemiologic surveys. While several methods would conceivably improve the N /C ratio, why did Takaki jump to adopt the Western style diet? Once bread was found to be unpopular, a substitute change to a diet of rice cooked with barley was resorted to right next year. Why he did not consider such possibilities to begin with? Did not he take for correct his theory of 'insufficient protein, harmful rice diet' from the start and then dash forward to confirm it? What does Takaki's silence signify in the face of such alternative theories as beriberi as a consequence of some infectious disease or a result of food poisoning? His attitude was as if to say that these theories were beneath notice." As Takaki's critics, e.g. academic researchers and Rintaro Mori, pointed out, he did not propose any scientific basis other than the N/C ratio as the mechanism of beriberi onset. Nor did he address any mechanism of prevention. Disease prevention could be implemented without firm evidence nor without knowing the disease's mechanism. Yes, a theory oftentimes becomes clear only later. In later life, however, Takaki published little by way of theoretical study.

Later, various replicate experiments with animals disproved Takaki's N/C ratio hypothesis. To think of it, N/C is merely a ratio of protein versus carbohydrates. Vitamin B_1_ content varies, depending on the food combination. It is no wonder that in some experiments the N/C ratio every so often turned out to be ineffective. It was lucky that Takaki's "improved diet" contained little rice so that his food combinations did not produce vitamin B_1_ deficiency. Given the lack of a theoretical mechanism, academic researchers continued their work on the assumption that the beriberi problem remained unsolved. Nor did an un-replicable N/C experiment help. Despite many intelligent studies on record, the situation at the time was one where no decisive conclusions could be reached even though cause of beriberi was close at hand. Researchers were hung up on the question of whether animal beriberi differed from that of humans. There might well have been some researchers who believed in Takaki's Tsukuba experiment and strove to validate it theoretically. Many in the field of hygiene had actually participated in the experiment. While Takaki viewed the N/C ratio as the cause of beriberi, the real cause was invisibly wrapped up inside a basket, the N/C ratio. Takaki appears not to have tried to unwrap the wrapper in order to see what was in it.

His achievement, eradicating beriberi, was evaluated highly. It received astonishingly great applause in the United States, the United Kingdom, and other countries during the era that followed the Russo-Japanese War. Prevention of beriberi greatly enhanced Japanese fighting power in winning that war's two naval battles. However, domestically, Takaki was long exposed to scholars' criticisms. For over 20 years he had to walk a thorny path. It is noteworthy that Takaki confined his efforts to Navy activities in the area of hygienic measures.

#### b. Beriberi Research in the Japanese Army

Beriberi was also a problem for the Army. Ishiguro, Director General of the Japanese Army Medical Service at the time, attached importance to funguses. He placed his hopes for finding the cause of beriberi on infectious agents of this type. Researchers at Tokyo Imperial University also continued to make fervent efforts along the line of infectious agent theory. Rintaro Mori, asked to evaluate Takaki's studies while in Germany to study military rations, immediately set to work on an "Outline of Japanese Military Rations". Based on this work, Mori's response to the question asked went as follows: "Given that the Army has in its ranks 10 times as many as the Navy, it is difficult for the Army to change its system of relying on rice. In addition to the difficulty of changing soldiers' eating habits the Army would also find it difficult to do Western style cooking. Nutritionally the Japanese Army ration is not inferior to that of European ones; therefore, there is no need for a change; problems, if any, can be investigated." Mori appeared to wish to be considerate to the Japanese attachment to rice-based rations. Upon returning home, Mori criticized Takaki's Tsukuba experiment as lacking in a control group. Therefore, in his opinion no scientific conclusions could be drawn. He also delivered a speech at a meeting of Dainippon Eiseikai (Greater Japanese Society of Hygiene) saying: "A non-Japanese diet makes a general lose his high ground". The gist of the speech was: recent European research found less protein is needed to maintain health than previously believed. With this result in hand, our present military ration does not differ much from theirs; therefore, Takaki's advocacy of a non-Japanese diet no longer has any merit." Shun'ichi Yamamoto pointed out that Mori's discussion was based on physiological findings and as such, was perhaps theoretically correct, but not relevant to beriberi prevention; it was akin to tripping up Takaki for a mere slip of the tongue. Another problem was that Mori did not have any data of his own to present. Mori later on had six soldiers eat three types of diet for eight days: polished rice alone, rice cooked with barley, and a Western diet with bread and meat. The judgment made in subsequent health checks as well as fecal/urine test was that the polished rice diet was best, rice and barley diet second best, and Western style diet the last. This attempt at a short-term physiological examination hardly had much significance in the pursuit of the cause of beriberi. Adding Mori's opinion, in 1890 Director General of the Army Medical Service Ishiguro presented a report on military rations to the Minister of Army stating that best was the rice only diet. As a member of beriberi infection group and relying on Mori's report, Ishiguro would not change the army rations. Naturally, beriberi incidence in the Army did not decrease. Great regional variance in the incidence of beriberi in the Army was a hindrance in grasping the general underlying rule to beriberi occurrence. One factor was the ease of obtaining food stuff on land as well as differences in availability in different areas. However, high beriberi incidence was an important problem for the Army also. As described above, the Osaka army unit managed to reduce beriberi incidences by implementing a rice-barley diet around the same time as the Navy did. As armies stationed in other areas began to understand the effectiveness of this barley mixed diet, this type of diet came to be proposed in some army districts. However, the Director General kept ignoring such initiatives and continued to adhere to polished rice.

Once the Sino-Japanese War began, the Army suffered a tremendous number of beriberi patients, while the Navy hardly had any. In 1894 during the war, a proposal by Yorinori Toki, local director of the Medical Division with the Japanese force in China, proposed to chief commander Ohyama the necessity of adopting a rice and barley diet. The proposal failed because of opposition from Headquarters. During the campaign in Taiwan immediately following the Sino-Japanese War, Toki supplied a rice and barley diet to soldiers, in consideration of the land's weather and climate. Upon hearing Toki's act, an enraged Ishiguro issued an order stating he would not permit a scientifically groundless rice and barley diet. Toki repudiated and responded in writing. His introductory remarks were: "... As hundreds of questions arise from the official directive, this officer dares to expound his opinion because of worries about the future. This is a candid opinion whose main purport is based on far reaching policy. Please understand that this argument is unlike the controversial disputes of academics." Following the above, he copied the texts of the directive, and then went on to the effect: "... To imagine the outcome of this directive makes one's hair stand up in horror... This officer hopes from the bottom of his heart that you Honorable Director General with a distinguished service record on whose shoulders a heavy responsibility rests would not lose his perspective by listening to the words of a small-minded man (meaning Mori) who envies another person's accomplishment..." Toki's act was quite extraordinary. However, his petition was not accepted, and as a consequence, many became casualties of beriberi in Taiwan, too.

When Masanao Koike became Director General in 1899 succeeding Ishiguro, who retired, he recognized the usefulness of the rice-barley diet. He gave an address of instruction to the effect that he would not prohibit the adoption of a rice and barley diet, even in the absence of scientific proof, as long as it was beneficial in practice. The following year the beneficial effect of this rice and barley diet was recognized officially. The diet thus came to hold a dominant position. Mori, however, still published a paper in 1901 titled "Reduction in beriberi with barley mixed with rice?" He argued: "The decrease of beriberi incidence observed in Japan and Indonesia in recent years appears to be a natural trend. I think the trend may possibly be due to some unknown cause." However, his bearish remarks at the end of his article also said: "My words are only isolated ones and do not flow with the mainstream... Reporters should not abstract this article but should reprint the entire text." With such remarks, Mori might as well have admitted that his theory was mistaken without so saying. As it happened, the polished rice ration was still in effect with the Russo-Japanese War's start. War dead due to beriberi amounted to over 27,800, 3% of all troops and the highest on record over the history of the world's wars. The number also represented one quarter of the total sick and injured during this war. When finally the Army was forced to shift to a diet of rice mixed with barley, the war was already close to its end.

The lack of consensus as to the cause of beriberi that persisted even after the Russo-Japanese War led the Army to form a provisional committee for beriberi investigation with Mori as its chairman. At the time he was the Director General. Minister of Army Masatake Terauchi stated: "While the proper nature of beriberi research belongs to academia, the Army has long been fighting the disease and possesses abundant research materials. I hope that this committee will aim to study the causes and treatment of beriberi. I also am a patient with beriberi who eats barley mixed rice. When I requested barley for my soldiers' diet, Baron Ishiguro, as well as Bureau Chief Mori not only opposed me, but also put me through a grilling questioning... This committee was set up to elucidate the cause of beriberi, the source of so much of the Army's pain..."According to Shun'ichi Yamamoto, Mori alone was put on the dock because many of his senior officers/fellow proponents of the infection theory had already retired. Going into 1924, the committee met 29 times. As its first project, the committee dispatched an inspection team to Indonesia where beriberi was rampant. Information was exchanged on spot. At home discussions were held covering a wide range of fields, e.g. fundamental, clinical, and epidemiologic problems of beriberi. At committee meetings, Army researchers presented a series of experimental and epidemiologic study results that showed that beriberi resulted from a polished rice diet. Thus, the theory of polished rice as the causal factor of beriberi gradually became firmly established. The committee's final cooperative project was a well-designed human experiment executed over 1923-1924. The project's overall conclusion was that "the main cause of beriberi is vitamin B_1_ deficiency." Mori passed away two years prior to the delivery of the conclusion.

In his will, Mori abandoned all honors bestowed during his lifetime, and stated: "I wish to die as a native of Iwami (a part of Shimane Prefecture)... No other letter should be etched on my epitaph than Rintaro Mori." Shun'ichi Yamamoto related that Mori shouldered all the responsibility alone. On the occasion of the death of Emperor Meiji and that of General Nogi who followed his Emperor to the grave, Mori penned a fictional story "The Will of Okitsu Yagoemon". It contained a tale of a samurai who perceived a contradiction in his master's order. In order to fulfill it, he had to cut down with a sword a colleague who had advocated reasonableness. Upon the master's death, Okitsu who had fulfilled his lord's command followed his master to the grave. Mori also published other stories on this theme, which may give a glimpse of his philosophy of life. With his studies Mori had absorbed modern medicine's theoretical system, believed in it, and behaved accordingly. However, he may have felt ashamed of having made a mistake that he thought followed from just that system. He had had no doubt that his superiors were pleased by his work. He believed that he had made no mistake in continuing to support Japanese rice as the Army ration. After 1900, however, even he himself came to vaguely suspect some special substance as the causal factor of beriberi. Mori graduated from college at the age of 19 years, was selected to study in Europe, where he distinguished himself and was unrivaled in logical thinking as well as in discussion. A brilliant person who early rises in the world tends to be excessively self-confident and to assume command. At the time of Mori's stay in Europe, however, medical theory there was not well developed either. Medical research on human groups in particular was still in its infancy. Mori himself still lacked experience. He held an over-optimistic outlook on his status as a researcher and on his future career. Research on the existence of special substances other than basic nutrients and the possible implications of their deficiencies on health problems progressed in Europe only after Mori's return to Japan. Thus, while acquainted with epidemiology, he appears not have understood its substance well enough. The fact was that, at the time, the majority of Japanese researchers, Mori among them, espoused a determinism that slighted probability theory. This too might have blunted the sharpness of his judgment.

In 1910, Umetaro Suzuki discovered oryzanin from rice bran. In 1912 Funk named the substance a vitamin. However, many authorities did not recognize the curative effect of oryzanin because everything about beriberi could not be explained by vitamin B1 deficiency alone. The effect of oryzanin on beriberi was only fully recognized 1924-1925, when finely tuned basic, clinical and epidemiologic research over 10 years by Professor Junjiro Shimazono of Kyoto Imperial University proved it definitively.

The beriberi problem is a good example for demonstrations of how medical research is and should be done. As it turned out, the epidemiologic research method played a major role in the process that led to the settlement of disputes. However, decades had to pass to reach conclusion because of the extremely low recognition of probabilistic research methods. In Japan, determinism has traditionally been given importance at the expense of probabilistic research. This tradition is still reflected in the status of epidemiology within present day Japanese medical research. Therefore, the beriberi problem remains the problem of today's epidemiology.^7^^-^^17^

### B-1. Geo-statistical Studies of Cancer

In 1900, the number of people who succumbed to cancer in Japan was about 20,000, or about 2% of the overall mortality. It was considered to be a major cause of death among the aged. The etiology of cancer was totally unknown and there were no methods of treatment; however, the study of cancer had already started. Research was not limited to experimental studies: attempts were being made in statistical studies and clinico-epidemiologic observations were under way on patient cohorts and in certain specific communities. The present author has already introduced outlines of these studies. In this chapter, their approaches, methodology and results are summarized together with their standings in the epidemiologic studies in Japan.

Professor Akira Fujinami of the Department of Pathology, Kyoto Imperial University held that environmental factors play a significant role, as well as the constitutional disposition in the etiology of neoplasms. In Europe, it was believed that regional differences in cancer incidence represent differences in the environment; and in the latter half of the 19th century, researchers tried to explain the patterns of cancer incidences through a geo-pathological approach. Fujinami was interested in this approach and became a member of the International Society of Geo-Pathology that was established in Switzerland in 1900. For a preliminary study, he and his friend, Dr. Sunao Nakarai, evaluated the accuracy of clinical diagnoses of cancer from autopsy records and ascertained the accuracy of mortality statistics. Subsequently, they surveyed mortality in several cities, towns and villages of Kyoto, divided these areas into 3 zones by mortality (high, intermediate, and low); then they compared such factors as natural environmental conditions of the residence, manners and customs, diet, income, occupations and work conditions between the zones with high and low cancer incidences. Summaries of their studies were introduced at a meeting of the Japanese Cancer Association in 1907 and 1908. Later, Nobuyoshi Suzuki, a disciple of Professor Fujinami, under the tutelage of the latter, conducted a multi-phasic epidemiologic study (Table 2) to detect environmental factors related to cancer pathogenesis. First, he computed the cumulative cancer mortality for the past 10 years at all cities, towns, and villages of Kyoto, classified these into high, intermediate, and low cancer incidence areas, and compared them to observe whether there was any correlation with other factors that were suspected to be associated with cancer pathogenesis. Specifically, the annual variations in mortality were moderated by the cumulative cancer mortality and the high and low incident areas were compared in an attempt to detect the characteristics of environmental factors. This exemplifies the epidemiologic study by contrasting areas. Suzuki spent 5 years conducting the study, analyzed and compiled the results and published the fruits of his labor in a 3-part discourse of about 200 pages. In addition, he conducted a similar study in Shiga Prefecture, east of Kyoto. Ten years later, identical surveys were conducted in Nara and Kagawa Prefecture by other students, also trained by Professor Fujinami. Professor Naosuke Hayashi of the Department of Pathology, Aichi Medical College, who was another of Professor Fujinami's acolyte, followed his beloved professor's teachings that extensive data should be collected in many areas over an extensive period before drawing any conclusions. He dispatched his students to 8 prefectures in central Japan (Aichi, Gifu, Mie, Shizuoka, Yamanashi, Fukui, Ishikawa, and Toyama) to conduct surveys. Assisted by advice from Nobuyoshi Suzuki, a method similar to the one described above was employed for this project. This research survey in 8 prefectures conducted by 8 groups required 10 years. A total of 8 articles were published between 1924 and 1936. All were extensive (200 to 250 pages), and took a long time at enormous cost.

**Table 2.** "Fujinami-Suzuki" study methods for malignant neoplasms summarized by the author.

1. Frequency and distribution of cancer deaths by town and village for 10 years in Kyoto and Shiga prefectures
2. Pathological features of cancer cases by site, age, sex, and area
3. Constitutional characteristics of cancer by sex and age
4. Hereditary factors by organ were based on familial aggregation
5. Comparison of clinical features with pathologic findings including metastasis
6. Comobidity such as other cancers, benign tumors, tuberculosis, and pneumonia
7. Causes of death
8. Cervical cancer and reproductive history of the patients
9. Occupation, working condition and cancer
10. Risk factors detected by comparative studies between high, moderate, and low mortality areas
Population density, climatic factors such as temperature, humidity, and rainfall
Topographic background such as high or low land, mountainous, lake, plain, and coast
Geology including surface soil and draining
Living backgrounds such as housing, drinking water, and sewage
Lifestyle habits such as eating, alcohol drinking, personal behaviors, and so on

In this study, the number of cancer patients with a low mortality was cumulatively computed for 5 to 10 years to minimize the data fluctuations; and a descriptive epidemiologic study was conducted. Those factors that might be related to the etiology were compared between the groups with high and low mortalities. To evaluate sample bias, the same study was repeated in different areas. This is a study to investigate the factors responsible for the development of cancers. It may be called the first epidemiologic study of cancers conducted in Japan. Suzuki, who conducted the survey in Kyoto and Shiga Prefecture, stated that his project progressed very slowly. Apart from individual studies that will be discussed separately, of the results of a study that encompassed 12 areas, Fujinami and Hayashi introduced the following conclusions on cancer mortality in Japan at a congress of the Japanese Society of Pathology:

1. From the evaluation of autopsy cases, the cancer diagnosis at the time of death was fairly accurate; therefore comparisons between regions were plausible. The mortality for the entire country was estimated to be 60 to 70 per 100,000 persons; however, one cannot ignore some regional differences. This mortality was lower than in the western world but the difference was not great. Incidence by organs involved differed by country and region, indicating significant involvement of environmental factors. In Japan, the incidence of stomach cancer was high, followed by those involving the esophagus and liver. On the other side, the incidence of lung, colorectal and breast cancers (in women) was very rare.
2. Familial clustering was expressed as 9.7% and within a same organ, 3.1%. Sarcoma was rare, the significance of which was under study.
3. Comparison among regions: In geographical and geological comparisons, the incidence of cancer was low in mountainous regions, that at high altitude, and with good drainage with no nearby rivers or lakes. The incidence was high in lowlands, areas with poor drainage; and low in dry highlands. A relationship with geologic conditions was noted.

The survey on etiological factors in the eight prefectures in central Japan detected common risks and some factors specific to the regions. Among these factors, the author selected those that are still accepted: they are:

4. Life style: Factors associated with a high incidence of cancer: limited vegetable consumption, gourmet diet, meat in the diet, excessive alcohol consumption, and heavy labor. Drinking water: water with high nitrate and nitrous acid (poor quality water). Beverage habits: coffee consumption associated with cancer. Esophageal cancer: associated with a habit of eating hot food. Often associated with habitual consumption of tea gruel. Others: Bathing in hot spas prevents uterine cancer
5. Correlations were noted between stomach cancer and occupational history, uterine cancer and reproductive history, and hepatic cancer and *Schistosomiasis japonicum*

#### Conclusion

Fujinami and Hayashi introduced their findings on the frequency distribution of cancer at academic meetings on separate occasions. Their data were convincing when the trend in cancer mortality was retrospectively estimated from 1950 when the statistics became relatively reliable. For a methodology also, diagnostic accuracy was assured through a comparison of the diagnosis at death and the autopsy finding before incorporating them in statistical computations. The study was not an only epidemiologic survey: basic findings from clinical examinations were compiled and analyzed to provide a comprehensive evaluation of patients' characteristics. The survey encompassed very many topics, such as environmental conditions including the natural environment that are sometimes suggestive of carcinogenic factors, living environment including housing, life style, occupation, and cultural elements. To detect possible carcinogenic factors, these conditions were compared among groups with high and low incidence of cancer. This approach is quite logical: the researchers probably believed that it is associated with less bias than case-control studies, while allowing for an understanding of local characteristics. Many of the factors thus detected were quite convincing and some have been reaffirmed by recent epidemiologic studies. Unfortunately, these studies by Fujinami and Hayashi were not introduced overseas.

For the shortcomings associated with the analysis of putative factors, comparing groups with high and low cancer mortalities is difficult when attempting to quantify the characteristics of the population, hampering the interpretation of the results. The methods used in surveying and measuring, classification, and quantification were unsophisticated. Statistical analysis was still in its infancy and there was no appropriate testing method available. However, the studies encompassed all of the methodologies basic to modern epidemiology. One can learn much from them even now. Unfortunately, they have been neglected over the years. Fujinami was well aware that a disease develops within a background of many factors; but he simply stated that these geo-statis-tical surveys should be conducted over many areas and be repeated multiple times before arriving at a hypothesis. Such statements represent his humility as a researcher. His studies required much time and the publication of this work (except that on cancer mortality) was delayed. Because of his sudden, untimely death, no overall conclusion was made public. Hayashi also resigned from his position at the university in 1931. Neither did he touch on a conclusion of the study except for cancer mortality. The interest in Japan in those days was on experimental cancer research: reflecting on the academic level at that time, geo-pathological studies did not attract much attention. As stated earlier, determinism ruled the academe and a probabilistic approach was not appreciated in those days.

Later, the research method adopted by Fujinami and Suzuki was utilized in search of the etiology of other diseases. It had a significant effect on epidemiologic studies after 1945.^18^^-^^23^

### B-2. Nationwide Survey on Cancer Patients and Estimation of the Incidence

Mataro Nagayo planned the compilation and analysis of the data on cancer patients who were treated at major hospitals throughout Japan for several years, starting in the second half of 1920. The details of his study were published in a special issue of the medical journal, "Gann" in 1935.

His study included changes in cancer mortality in Japan stratified by organ (examined at 5-year intervals between 1915 and 1930) and the percentage for each organ involved, comparison of the data with those from overseas and age and sex distributions. From 1929 through 1932, he sent a form to university hospitals and large hospitals in the country and asked them to fill out details on their patients. The cases reported included 903 from general hospitals; 5,395 from the departments of internal medicine, 6,501 from the departments of surgery, 3,400 from the departments of gynecology and obstetrics, 1,307 from the departments of otorhinolaryngology, 375 from the departments of dermatology and urology, and 20 from others (all from university hospitals); 1,654 from hospitals specializing in gastro-enterology; and 1,158 from the treatment section of the Cancer Research Institute. When stratified by organ, a total of 20,782 cases were compiled. The survey period varied among facilities and were not necessarily uniform. A similar survey was conducted by Fujinami but one of this scale had never been conducted. It appears that the bias was not small because of the size of the data source.

The autopsy data on cancer patients obtained from the Department of Pathology, Tokyo Imperial University were divided into two groups by period: from 1894 through 1914 and from 1915 through 1932 (812 and 767 cases, respectively). They were stratified by gender and age and analyzed for their distributions by organ or facility where the patients had been cared for. The data were further compared against those from foreign cities and facilities to discover international differences or similarities. To evaluate the accuracy of clinical diagnosis, the failure in detection while the patient was alive was 14.02% and errors in pinpointing the cancer site, 2.89%. These data called for caution in interpreting the statistics for cancer mortality.

Based on the comparison of these 3 large cohorts of cancer patients, the following were stated definitively: the frequency of cancer incidence was low in Japan but did not differ much from that reported overseas; cancer mortality appeared to be on the rise but the increase was not particularly notable; when stratified by organs involved, the data differed greatly from those from overseas; and from the incidence of cancer - by age, sex, physiopathology, and life style (such as food habits) - all have a significant bearing on the development of cancer, for which the author explicitly stated that it was possible to prevent cancer. The fact that a pathologist introduced a comprehensive finding from epidemiologic research is highly appreciated.

In addition, he introduced similar geo-statistical surveys in China, Korea, and Taiwan.^6^^,^^23^^-^^25^

### C. Clinical and Geo-pathological Studies of Rickets in Toyama Prefecture

Descriptions of rickets have been in existence for many years in foreign literature. Already in England in 1645, rickets among children was described. In Japan, Professor Baelz of Tokyo Imperial University declared that there is no rickets, which was widely believed then. Earlier, in 1888, however, an incidence of rickets had been reported in Sendai. In 1901, a number of cases were reported in the Hokuriku area (Toyama, Ishikawa, and Fukui Prefecture); and between 1893 and 1895, cases were reported one after another in the Kanto (around Tokyo) and Tohoku areas. In 1904, Akira Fujinami published his observation at an autopsy of a 10-year-old boy, which established the existence of this disease in Japan.

In 1906, the mortality in the Himi area of Toyama Prefecture was compared with the corresponding national figure to confirm the high incidence of rickets there. Furthermore, a survey was conducted on 2,723 residents in Kumanashi Village of the Himi area by local researchers, which detected 60 confirmed or suspicious cases of rickets. Subsequently, an investigation committee was organized in this village; and a request was made to Tokyo Imperial University to cooperate in the survey, which resulted in the formation of an ambulatory diagnostic service by physicians from the same university. The result was the confirmation of multiple incidences of rickets in a large area of Toyama Prefecture.

#### Survey by Masakiyo Ogata to Confirm Incidences of Rickets

Masakiyo Ogata, a gynecologist from Osaka having studied in Germany, attempted to investigate the cause, treatment and status of this endemic disease that often struck young boys and young women from a gynecological viewpoint. With the cooperation of a local university, physicians and legislative bodies, he conducted a clinical survey; and he also admitted some of the patients to his hospital to investigate the clinical features and the environmental factors that involved the patients, such as geological, soil, and meteorological features. He compiled these findings and published them in a book form. This publication is the compilation of a study of 305 patients, including 240 who were diagnosed and treated at Kumanashi Village (where a large number of patients were found), Himi area and 5 other villages; and 63 who were diagnosed elsewhere. The patients ranged from 1 to 15 years in age and the sex ratio of the cohort was 1 : 2, with females being predominant. The ultimate diagnoses were typical rickets and osteomalacia. Their symptoms, physiopathology, clinical course and outcome were observed and discussed in detail. Among the old patients, the characteristics were extreme pain when they moved; and among the infants, movement was minimized to avoid pain. These findings indicated that the main feature of the disease is body pain. Ogata also looked into the difference between rickets and osteomalacia and found that some cases to be a mixture of the two. The characteristic geological conditions in which these patients were found were the short duration of exposure to daylight: in addition, their homes were heavily shaded by trees and other objects, which further reduced their light exposure: their environment was also characterized by high humidity, low temperatures, and exposure to high wind. The housing was constructed with no particular design to let sunlight in; the ventilation was poor; and interior was dark; in general the hygienic conditions were poor. The infants were being raised under such conditions. A diet was low in food of animal origin and nutritional value. The quality of water was also poor. The parents of these infants engaged in harsh labor to earn a meager income. In particular, infants were confined in straw baskets (*tsubura*) and it was suspected that the restricted movement of their lower extremities hampered bone development. Although the direct cause of the disease was still unknown, Ogata believed that improving the structure of their homes and life style and upgrading their diets would prevent the onset of the disease. Because the immediate elimination of these putative factors was impossible, he recommended a solution in which that the inhabitants of these area could be moved to other part of the country: he made an appeal to the public to that effect. At that time vitamin D deficiency was not known as the cause of this disease.

Ogata conducted a study in which the patients were accurately diagnosed and the clinical and epidemiologic characteristics of the cohort were compared against those of normal individuals, although, few of these comparisons were made with quantitative data and the controls were not selected randomly. It should be appreciated, however, that Ogata accurately noted that the disease was not an infection but was closely related to environmental factors.

Later, the increasing number of cases of rickets and osteomalacia were reported in Japan. According to comparative observa­tions based on statistics for the 13 years between 1910 and 1922, cumulative mortality, stratified by area throughout the country, elicited particularly high figures for Toyama and Ishikawa Prefectures (more particularly in Himi, Takaoka, and Higashi Tonami counties). Rickets is basically a disease of infants and young children but some grow up, become pregnant and deliver children. This constituted a serious problem in some areas but throughout history it was intentionally concealed from the rest of society.

In Europe and the United States, an accurate diagnosis was given to patients with rickets; it was known that fish liver oil was effective for treatment; and rather than the infection theory for its etiology, the relationship between the disease and diet had been held vital. In 1917, E. Mellanby of the United Kingdom proved the efficacy of liver oil (in 1919, it came to be called vitamin D) in his experimental study. For the action mechanism of this liver oil, findings such as the interference of phosphorus and calcium absorption by phytic acid in the diet and the calcium-phosphorus ratio being a cause for the onset of the disease were reported (1921). All this information, belatedly introduced to Japan, was reflected in the research conducted in the 1920s.

#### Geo-pathological Studies by Renpei Shizu and Shokichi Ichioka

Renpei Shizu planned a geo-pathological study of rickets in the Himi area. Based on the geo-statistical method of Fujinami and Suzuki for the tumor research (introduced earlier), he conducted a survey for the entire area and compared the findings with those from other areas where no patients were found.

Using the data on 106 cumulative cases diagnosed in the Himi area during the 4 years form 1925 and those on 25 cases discovered by other physicians, he conducted a comparison between these areas where multiple incidences were noted and the control areas. When the putative factors were stratified by natural life environments and life styles, it was reconfirmed that a high incidence of the disease was in those areas with short sunlight exposure, higher amounts of rainfall, and higher humidity. The patients were often found in families with a maternal history of high parity and high incidence of stillbirths, with a clustering of patients among relatives. In the areas with a high incidence of the disease, the parents had been engaged in harsh labor; the fathers were much older than the mothers; there were many siblings with little age difference among them. The patient was the product after repeated pregnancies by his mother and suffered from an insufficient supply of maternal milk. He was left in an unsanitary, malodorous room that was rarely exposed to sunlight and tightly closed with poor ventilation. His protein intake from sources such as eggs and cow's milk was limited. His mother often suffered from beriberi. In this observation designed to compare regions, the results were similar to those reported in the case studies conducted by Ogata. The affected children indicated a poor growth pattern and various types of clinical deviations. They were anemic with a high leukocyte count; and their blood calcium, catalase and cholesterin levels were low.

For treatment, the effects of liver oil and exposure to artificial incandescent and ultraviolet lights had been indicated. In 1918, Hess introduced a therapy in which an artificial sunlamp was used. Shizu applied an adequate exposure of artificial ultraviolet rays and preparations containing liver oil to treat the patients diagnosed during his survey and reported an improvement in their symptoms. This indicated that his epidemiologic survey had been expanded to include the treatment and prevention of the disease.

Shokichi Ichioka conducted a similar geo-pathological study in the Higashi Tonami area in 1930. His report simulated Shizu's. He reported that the mothers were in a poor nutritional state and suffered from various complications. The patients were anemic and their serum phosphorous and calcium levels were low. When the households with and without the incidence of the disease were compared, the drinking water available to the former was high in ammonia, sulfates, nitrates, chlorine and organic substances. In other words, their water was of poor quality. Ichioka treated these patients with vitamin preparations containing vitamin D, which had already been discovered. He emphasized the adverse effects (such as anorexia, brownish skin deposition, and calcium deposits in organs) of 5 types of these preparations and warned against administering a large quantity because of the health damage might produce.

#### Adverse Effects of Vitamin D

In 1932, Yoshinobu Sugiura investigated the adverse effects of a large quantity of vitamin D in animal experiments, which had already been noted in Europe. He observed that administering an excessive dosage of vitamin D in an experimental animal resulted in anorexia, general lassitude, a decrease in motor activity, loss of luster of the fur, soilage of the body with feces, diarrhea, emaciation, resulting in death due to cachexia. He issued a warning based on these observations. He also stated that the extent of these ill effects depended on the person's age.

In addition, there were reports on the relationship between vita­min administration and calcium metabolism, in particular for calcium deposits on the aorta, renal epithelium and kidney. The original clinico-epidemiologic survey spurred the development of therapeutic research.

Clinoco-epidemiologic and geo-pathological studies were repeated on the disease, the cause of which had been unknown. It should be noted that the research was extended to include the evaluation of its therapy and the associated adverse effects, in addition to its incidence, physiopathology and clinical course. Unfortunately, these research activities were interrupted by wars and subsequently forgotten. After World War II, the disease re-emerged as a social problem in relation to Itai-Itai disease but the response to this problem was slow in coming. Regardless of the different cause of the disease, reference to the clinico-epidemiologic information that had been gathered in the past would have avoided the initial confusion.

Provitamin D was discovered in 1927, its crystalline form being called D2. In 1936, 7-dihydrocholesterol, a provitamin, was isolated from the liver oil of tuna, which was treated with X-rays and used as vitamin D3, an effective agent for the treatment and prevention of rickets.

#### Conclusion

Whenever a new disease was discovered, the researchers in those days responded by trying to define its characteristics and diagnosis: and by reviewing the literature from the western world, they were eager to define this entity. If its diagnosis was established, they conducted epidemiologic studies, in addition to studies from the viewpoint of clinical medicine and basic medical science, to find its etiology and mode of prevention. These studies were almost entirely financed by individual researchers, which made it impossible to include long-term follow-up studies.^26^^-^^33^

### D. Dropped Head Disease (Gerlier Disease or Paralytic Vertigo)

The 50-year History of the Japanese Society of Hygienic Study lists in the epidemiology section, among the major hygienic studies conducted before 1902, an 1888 report by Kenryu Nakano on dropped head disease observed in Aomori Prefecture; and the 1894 survey conducted by Kinnosuke Miura, Associate Professor of Tokyo Imperial University, on the same disease, also in Aomori Prefecture. This disease, a rare disease, had been studied later in Gifu Prefecture. It has been said that an epidemiologic survey had been conducted earlier and the author heard the name at a lecture given at a medical school in 1951. However, a search of the medical literature failed to produce any substantial data. I happened to find that my respected friend, Dr. Akira Takahashi, Professor Emeritus of Nagoya University, had published a short history of this disease. With his assistance, I was able to write this section. I thank him very much.

#### Report by Kenzo Nakano on Aomori and Iwate Prefectures

It was said that for many years there was an endemic disease called dropped head disease in the Tohoku Area. Nakano examined 283 patients over a period of 6 months in Kamikita and Sannohe areas in Aomori Prefecture and Ninohe and Kunohe areas in Iwate Prefecture. Based on his clinico-epidemiologic observations, Nakano described the disease as follows: symptoms included headache, a sensation of heaviness of the head, discomfort, and particularly among women, vertigo and on rare occasions, mild chills that precede the attacks. Then suddenly the patient experienced weakness of the neck and lower extremities: the head drops and his/her gait becomes unsteady. The attack ended within several hours; in addition, the disease included pupillary dilatation, dullness of sensation in the neck, disorders of speech, deglutition and mandibular movements, tinnitus, constipation, and diarrhea. This course of events ended in a few minutes to several hours; but occasionally it lasted for several days, several weeks or rarely, for several months. Climate or working conditions might have an effect. Normally, the patient recovers completely but might often suffer a recurrence. Recurrences might be fatal in aged patients. Treatment included oral administration of quinine sulfate, subcutaneous injection of camphor, internal and external use of mercury iodide, and the use of arsenicals and laxatives. These might be effective for a limited time. The cases were seen in isolated mountainous regions with high humidity, poor air flow, and inadequate exposure to sunlight. No incidents had been noted within 1 km of the coast. Patients were often seen among those living a simple life, in humble housing (a small house with a straw-covered floor), and engaged in heavy agricultural work. The incidence was high in the late spring and summer and low in winter. These were results of a clinico-epidemiologic survey.

#### Surveys by Yoshikimi Onodera and Kinnosuke Miura

During a survey on an endemic disease called "Nezumi-tsukuri" in 1894, Yoshikimi Onodera of Tokyo Medical University (the predecessor of Tokyo Imperial University) examined patients with dropped head disease at Sannohe area in Aomori Prefecture and conducted a survey of 28 patients with the cooperation of Kyoritsu Sannohe Hospital. In his report, he described the disease as follows: the disease striked anyone regardless of age or sex but most of the aged individuals and infants were spared. It occurred often in agricultural workers and it might strike two or more members of a single family simultaneously. It was not transmitted genetically nor from person to person, and it frequently recurred. The clinical course might be acute or chronic; occurrence was high in summer and fall; no recurrence was noted when the patient leaves the site where he/she fell ill and moved to a distant place; it developed episodically and almost no abnormal symptoms were noted between episodes. Fatigue, emotional unrest, indigestion and hunger tended to trigger attacks, which were not likely to occur when the patient was emotionally stable. Attacks are suppressed when the patients eat a large meal or consume chicken eggs; the severe attacks never threatened life: the major symptoms comprised of a sensation of hunger, depression, photophobia, diplopia, blepharoptosis, paralysis of the lips, paralysis of the masticatory and deglutition muscles, frequent blinking, reduced visual acuity, movement disorder involving the extremities and tongue, head droop, weakness in the pelvic region, tinnitus in rare cases and an abnormal taste sensation. The similarity to malaria was noted. The report by Nakano resembled that of Onodera although their studies were conducted independently.

Kinnosuke Miura conducted an academic survey by order of the Ministry of Education. He diagnosed 69 patients in 6 counties in Aomori and Iwate Prefectures and compiled the results in 3 reports in 1894. He also published the results of his work in German. He stated that dropped head disease was generally identical to Gerlier disease that had been introduced in Switzerland in 1886. Through Miura's report in German, the name, dropped head disease, came to be known throughout the world. The results of his clinico-epidemiologic survey were generally similar to those of Nakano and Onodera. It was concluded then that the disease was paralysis of the deep nuchal muscle.

#### Dropped Head Disease Found in Gifu Prefecture and a Geo-pathological Survey

In 1908, Kizo Watanabe reported on 7 cases of dropped head disease in Anpachi area, Gifu Prefecture and described its geological characteristics.

Between 1915 and 1923, Saburo Inoue engaged in a survey of 34 families or 119 patients with this disease who resided around Gifu and Ogaki Cities and investigated the disease by employing a geo-statistical survey method, such as that used by Fujinami and Suzuki. Inoue's survey method is summarized below:

1. Discovery of cases, evaluation of the disease as an independent entity and its differential diagnosis
2. Geographical and temporal patterns of incidences and clustering of cases
3. Symptoms, clinical course, physiopathology and prognosis of patients
4. Epidemiologic features of the patient cohort
  Geographical and geological features: Terrestrial conditions, terrain, mountainous region, plain, areas around rivers, aridity, and humidity
  Meteorological features: weather, rainfall, temperatures, and air humidity
  Residential areas: housing structure, exposure to sunlight, ventilation, sanitation, the presence of domestic animals
  Life style: diet, drinking habits, quality of drinking water, occupation, industry, working conditions, and psychological stress
  Medical history: past illnesses, current medical conditions, preceding illness, delivery, child nursing, and sibling and family history

Enormous amounts of time and expense were spent on this survey. In search for etiological factors, the areas where multiple incidences were noted and were compared against the control areas. For the clinical investigation, each patient's asthenic state was experimentally studied. In addition, the effects of drugs on the disease were observed. Clinical examinations included an X-ray examination, blood pressure determination, blood chemical analyses and analyses of gastric juice, urine and feces. Experimental studies on fatigue phenomena and electrical responses of the nerves and voluntary muscles were also conducted. A detailed differential diagnosis was conducted for the discussion to define the entity of this disease. His report, accompanied by maps, was composed of 179 pages.

The epidemiologic features of this disease are summarized as follows: its incidence was highest in January, followed by June and October but it occurred less frequently in April, May, and July. Among agricultural workers, the incidence was limited to those in the middle class and their families. It was rarely seen in the wealthy class. The significance of housing, which was described in detail earlier, was found to be unrelated to the incidence. An isolated incidence was rare: in most instances, several or all of the family members in a household developed the disease over a period of 2 to 3 years. Severe symptoms lasted from one to 10 days, most frequently 2 to 3 days, and the patients recovered spontaneously. None was found to be fatal. The disease condition exacerbated temporarily after consuming a large amount of food, such as rice cakes but eating raw eggs eased the symptoms. The disease was unrelated to a meager diet. The onset in those under 10 years of age was 30.9% but it was very rare among those over 40. Body constitution or the nutritional state was unrelated to onset. For factors provoking the illness, lack of sleep, physical and psychological stress from work, and changes in one's work situation were cited. Parasitic diseases were found to be unrelated. These findings were generally similar to those given by Nakano and Miura. Inoue did not refer to the prevention of the disease: the number of cases in his study was limited and no analytical studies followed.

During the Meiji Era, there were reports of cases found in Tokushima and Niigata Prefectures; however, none were found in the Showa Era. It is suspected that the disease ceased to exist in the modern era.

In Europe, Gerlier stated that the occurrence of this disease coincided with the epidemic of Behçet disease, strongly suspecting cryptogams infection for its etiology; however, there are no studies to follow up this theory.

It should be noted that in the Meiji Era, active investigations on disease status and their etiological search were made even for rare, geographically localized diseases.

#### Recently Noted Dropped Head Disease

Although different from the original dropped head disease, patients with the head-dropping symptoms in association with other diseases have recently been reported in Europe and Japan. In the latter, the associated diseases are: facioscapulohumeral muscular dystrophy, hypothyroidism, myasthenia gravis, hypokalemic myopathy, and central nervous degenerative diseases (such as multiple system atrophy, progressive suprabulbar palsy, amyotrophic lateral sclerosis and others). From the similarities to these diseases, one may explain the etiological mechanism of dropped head disease that plagued a number of Japanese earlier. On the other hand, the disease conditions undergo changes with time. The relationship between environmental changes and the development of diseases may be an issue to be faced in future.^34^^-^^41^

### E. Nutrition and Epidemiologic Survey

Research on energy metabolism began in the 18th century, and by early 19th century evolved into research that linked metabolism to breathing. Following the separation and naming of three major nutrients by William Prout of the United Kingdom in 1927, physiological as well as biochemical research on nutrition progressed. Research on nutrients and digestion also progressed over the latter part of the 19th century. At the same time, the important role of minerals in nutrition also came to be understood.

Since the 1880s Japanese researchers who did their studies either in Europe or in the United States began their research based on ideas about nutrients or energy they had learned abroad. Vitamins were still unknown in Japan during the1880s. However, the importance of nutrition in peoples' lives had, by that time, long been recognized. Consequently, several field studies had already been carried out: an 1885 nutritional survey on prisoners by the Tokyo Drug Laboratory; an 1887 survey by the Tokyo Hygienic Laboratory on students and store employees. Over 1887-1888, Jiro Tsuboi, student at Tokyo Imperial University (later Professor of Hygiene) together with others, surveyed the food and nutrition of students living in dormitories, lodgers, clerks, and laborers. These surveys tried to examine the relation between health and nutrition from the perspective of hygiene.

In 1887, Yoshizumi Tawara made available tables of food composition and nutritional requirements. In 1909, the Tokyo Hygienic Laboratory published an "Analytical Table of Food, Beverages, and Nonessential Food Items". In 1931, the Nutrition Research Institute of the Ministry of Interior prepared a "Comprehensive List of Japanese Food Composition" containing 1,045 food items. Thus the infrastructure necessary for scientific surveys was being readied.

Owing to the beriberi studies described in the earlier part of this article, nutritional problems attracted the attention of the Japanese general public as well as that of the medical world. The Japanese Army as well as the Navy had placed special importance on research addressed to the military. As was described above, the Army had dispatched Rintaro Mori to Germany. While there, Mori had authored a noteworthy work on the military ration, i.e. the comprehensive work "Outline of Japanese Military Rations" that he had composed as well as a "Report on an Examination of Military Rations", authored in Japan upon his return. Kanehiro Takaki of the Navy had hypothesized that beriberi was a consequence of biased nutrients. Takaki's own experimental study aboard naval ships while on a long distance cruise had been a success. Beriberi was eradicated from the Navy through improvements made to rations. From around mid-19th century, research in Europe had begun to direct its interest toward health disturbances caused by lack of certain non-nutrient substances. This line of research led in the 20th century to the discovery of vitamins. Stimulated by the beriberi disputes described above, nutrition research along these lines began to gain force in our country as well. Young talent, stimulated particularly by Umetaro Suzuki's discovery of oryzanin, became attracted to nutrition research and began to apply physiological and biochemical methods to their work. Meanwhile, physicians and scholars of hygiene also began to examine relationships between nutrition and health disorders among the populace and laborers, as described previously. However, the broader class of medical scientists tended to slight nutritional research as not being one of the pure sciences. Professor Junjiro Shimazono was an exception. He was a physician who stressed not only basic and clinical medicine but also valued an epidemiologic survey that seeks verification over groups of humans. He had proved that the causal factor behind beriberi was a diet of polished rice through his comprehensive surveys at spinning mills and prisons, sites of many beriberi cases. Among the world's medical associations, the study of nutrition had long been considered a minor branch.

In the present article, the author would like to introduce Tadasu Saiki. Saiki demonstrated the importance of nutrition through his research in experimental, clinical, and preventive medicine. He had linked it to human development, maintenance and enhancement of health, as well as disease prevention. While laying the groundwork of the science of nutrition, he strove to nurture our human resources. In this way, Saiki further developed the methods of the scientific nutritional survey of human populations.

After graduated from the faculty of medicine, Saiki studied biochemistry. Later while doing research work at the Institute of Infectious Diseases in Tokyo, he discovered radish diastase. He also examined nitrogen balance in beriberi patients. Saiki studied physiology as well as physiological chemistry under Professor Russell H. Chittenden of Yale University and acquired an academic degree (there was no nutrition course at Yale University at the time). Upon his return to Japan in 1914, he established a private nutrition research laboratory and engaged in practical nutrition survey/research, as well as in practical nutrition education. He expressed his novel aspirations as follows: Nutrition is the basic underpinning of society. Nutrition determines health, economy as well as morality; no life is possible without nutrition. Nutrition research should not limit itself to its theoretical aspect, but should also study its practical aspect scientifically; this is the way to realize glorious human life that includes consumption as well as production. His science of nutrition preceded biological study. His method was first to focus on a phenomenon in an organism, pondered its significance, and then to proceed to study its scientific composition. He also attempted to establish practical theory through epidemiologic research and have it contribute to the prevention of diseases. He had a penchant for new ideas. One such example was a field study among the general public in parallel to basic experimental research at his private nutritional laboratory. The exhibit at the Taisho exhibition of a sample healthy daily diet for a Japanese person over one day was his laboratory's attempt to enlighten the general public.

Saiki proposed a different combination of Chinese characters to stand for the word 'nutrition' in the Japanese language – a departure from the then current usage. He also created Japanese technical words related to nutrition e.g. unbalanced diet, nutritional counseling, rice with germ. These words came to be widely used both in nutrition research and in practical nutrition education. He emphasized the importance for children's development of a school lunch. Beginning in 1923, the year of the Great Kanto Earthquake, the merits of the school lunch began to be understood and thereafter the practice became popular. He established a professional school of nutrition to train specialists with the purpose of securing human resources for the implementation of his various projects. All these projects were in Japan novel in kind. Stimulated by the discovery of a string of vitamins, researchers' as well as the public' interest in nutrition grew. As a result, the Ministry of the Interior in 1920 established the National Institute of Nutrition at Saiki's suggestion and appointed Saiki its director. Saiki closed his private nutrition laboratory, because he thought that activities were necessary to promote nutrition at a national level. The institute consisted of divisions for basic research, applied research (food problems, cooking and cooking implements, child nutrition, etc.) as well as an investigative division (cooking, statistics, historical materials, training program, exhibition, public relations, etc.). The Institute aimed to devise nationally applicable nutritional measures through epidemiologic surveys. The Japanese Nutrition Association, established by Saiki, evaluated the expansion of the school lunch program and its effects on children's health and development. He also created a school of nutrition to train nutritional technicians (later called dietitians) to propagate the nutrition movement based on results of scientific research. Nutrition thus prevailed as a branch of practical science and also came to be considered in research on diseases. In Japan, "Nutrition" was recognized as a science in 1934 under the name of dietetics. In the same year, the Japanese Society of Nutrition came into being, the first in the world to be established as a professional nutritional society.

Saiki's research activities were wide ranging and were internationally recognized. Advancement of research that integrated modern dietetics with epidemiologic surveys was achieved with this background.

Now, the author would like to present an example of a typical nutritional/epidemiologic survey as a supplement to the section E. The following is based on a copy of a paper given to the author by the late Professor Emeritus Hajime Miura of Kumamoto University.

In 1930s Professor Un'ichi Miura and others of the Department of Hygiene, Manchurian Medical College, began a survey of Manchurian (Northeast China) farmers' life habits, eating practices, nutrition, and health. The survey continued into 1944. The first field survey took place in Mukden (today's Shenyang City) over 1928-1930. The survey encompassed residents in 26 occupational categories and social classes (upper, intermediate, and lower). The total number of reported cases was 1,370. Variety and quantity of consumed food (staple food and side dishes) in this survey were compared and examined for each nutrient. Another survey on the eating habits of farmers in various parts of Manchuria compared results from a nutritional perspective. In addition, longevity, mortality, and frequency distributions for various causes of death were surveyed and compared; great disparities were observed among social classes. Also observed was the influence of improved dietary practices on mortality and on productivity. These results were compared to data gathered from Japanese inhabitants living in Manchuria or in Japan proper. It is noteworthy that relations between nutrition and geographic/geologic factors as well as nutrition and physical development were also documented. The studies strongly influenced post-1945 research in Japan.^15^^,^^16^^,^^42^^-^^44^

### F. Effects of Dietary Practices on Stroke and Longevity: Epidemiologic Survey

Shoji Kondo, who specialized in hygiene at Tohoku Imperial University and later became a professor at the university, studied the effects of ultraviolet radiation on health. He recognized the effect of sunbathing on school children, thus pioneering a practical research field of school hygiene. Kondo set out to find causal factors behind short life expectancy among residents of the Tohoku region. Examining national mortality statistics, he identified places with high and low numbers of over 70 year olds; he called the former long-lived villages, the latter, short-lived villages. He began to visit the actual sites one by one, seeking factors responsible for the difference between the two types of villages. When he surveyed short-lived villages in Akita Prefecture, he found that stroke lowered villagers' lifespans and suspected that an unbalanced diet, e.g. polished rice staple, was the culprit.

He visited short- and long-lived villages nationwide repeatedly exploring relations between diet and lifespan. Meanwhile, he published works on links between diet, work, life style and life expectancy and stroke, based on observation taken during those visits. At the 43rd subcommittee of the Japan Society for the Promotion of Science in 1945, Kondo reported on a number of factors increasing the number of deaths after stroke: (1) living in cold regions: (2) rice farming; (3) eating large amounts of rice; (4) excess intake of salt; and (5) heavy drinking of alcohol. On the other hand, the risk of stroke and deaths after stroke was low in fishing villages where seaweed was a common component of the diet. While objections to his survey methods were later raised, e.g. problematic selection method of interviewees or questionable reliability of listen-answer style interviews, the published results themselves appeared to have been to a large extent correct. In the author's opinion, Kondo's walking-shoe style epidemiology laid the foundation for epidemiologic surveys of cardiovascular diseases too. He continued his survey visits to towns and villages all over the country to confirm his hypotheses, corrected his mistakes, and endeavored to prevent early death through improvements in people's life style. Even before 1944, he also preached the importance of age adjustment in studies of mortality.

From 1930 to the 1940s, the third leading cause of death in Japan was stroke; for middle-aged or older, it was the leading cause. In 1941, a multidisciplinary research team was set up to study stroke incidence in its pathological, causal, clinical, and statistical aspects. Epidemiologic surveys of stroke other than Kondo's included those commissioned by the local governments of Chiba and Osaka Prefecture. Follow-up studies by life insurance companies also published important findings. While the expression "epidemiology" was not used in the studies, these in fact were epidemiologic surveys that employed survey methods unique to each. Epidemiologic studies of cardiovascular diseases after World War II evolved from foundations laid by the above described works.^45^^-^^52^

## CONCLUSION

The author presented an overview of some early Japanese epidemiologic studies. Some of the articles may not be treated as epidemiologic studies in a strict sense but all were studies on etiologies and etiological factors, with patient cohorts, population groups and local populations for subjects. Thus they were included so that these subjects would be covered from a broad viewpoint. Through these studies, one can understand that the early researchers endeavored to detect the etiology of diseases over a wide spectrum. Many pages were devoted to the topic of beriberi. The studies on this subject marked the dawn of epidemiologic studies in this country and one can gain many lessons from the details of the arguments that took place in those days. There are other epidemiologic studies that the author would like to present here, but this will have to wait for another occasion.

As indicated amply in the body of this presentation, from 1880s, clinical investigations and the resultant treatment, as well as basic medical and epidemiologic studies, were conducted on diseases of frequent incidences, endemic diseases and even those with unknown etiology that caused much pain to patients and their families. The prevailing attitude of the researchers was to conduct a comprehensive study by utilizing statistical and geo-pathological approaches so that they might formulate methods to prevent these diseases. The studies were led by clinical physicians with the participation of pathologists, hygienic specialists, and those specializing in basic medicine. Their approach appears to have been far more practical than the current method in which the projects are divided vertically for each specialty. As a methodology for epidemiologic studies, these may be criticized as being unsophisticated; but they succeeded in detecting many risk factors that were helpful in medical practice, thus contributing much to the welfare of the public. The author believes that they offer many valuable lessons to aid in modern epidemiologic studies. They should have been valued highly on the international scene in those days but unfortunately only a small part was published in foreign languages. Nutritional studies were included in the present selection of articles because we are now at the stage when public health and disease prevention cannot exist without these studies; yet, they have been ignored far too long.

In the field of epidemiology, little attention had been given to probabilistic research; and universities did not even have an independent course on this subject. If each outstanding epidemiologic study had been linked horizontally and evaluated comprehensively, epidemiology and related medical studies unique to this country would have developed. The absence of a system to incorporate epidemiology into mainstream medicine, together with the delay in the development of this discipline, contributed to the slow refining of medical science. Most of the funding from the epidemiologic studies cited here came from non-governmental sources. It is unfortunate that the continuity of these studies was lost due to a lack of financial support.

Since 1930, research in statistics advanced gradually but steadily in western countries as evidenced by topics such as the selection of a parent population, setting of diagnostic criteria, classification and quantification of etiological factors and selection of controls. Aided by the refinement of statistical tests, this discipline has come to be applied actively to medical science since 1945, contributing much to the development of modern medicine. On the other hand, in Japan, statistics was long neglected in the academic medical circles, leading to a delay of expansion of clinico-medical studies.

In Japan, the epidemiology section was created in the National Institute of Public Health in 1938, but World War II broke out after that. Researchers were sent to the battlefields; research materials became scarce; and many valuable records were lost during air raids. Social upheaval following the end of the war delayed the progress in epidemiology more than in other medical fields. Compared with the dramatic progress in the western world since 1930, the delay in this country was evident.

## DIVERGENCE OF STATISTICS FROM MEDICAL SCIENCE (ADDENDUM)

When diseases began to spread repeatedly in the country in the 19th century, scientists turned to statistics to analyze the frequency distribution, trends, and characteristics of diseases. At the same time, the sociological and economic effects on diseases were clarified, adding directionality to medical research. Epidemiology has its origin in a discipline to give answers to questions arising from clinical medicine: therefore, it has a close relationship with statistics. Initially called national population studies, statistics was used to comprehend the social and economic status of diseases, and soon thereafter it came to be used in the field of medicine, especially to provide a realistic understanding of prevalent diseases that had some social impact. Statistics on population problems and mean life expectancies also came to play important roles in medicine, which was cited as an important area at the International Statistics Conference that was held in Brussels in 1853.

It has been said that Masamichi Tsuda and Amane Nishi, students who were sent to the Netherlands by the Tokugawa Government in the 1860s, studied statistics together with politics and law. They played active roles in the newly established Central Government during the Meiji Era. In 1872, the Government created a statistics office within its organization to start related activities. A course in statistics was offered at the Daigaku Nanko (later Tokyo Imperial University). Thus a fairy large number of students were trained to be professional statisticians.

Census taking based on the family register system and a survey on population dynamics were launched. As stated earlier, the statistical yearbook (later Japan Statistical Yearbook) was published annually starting in 1882. In the field of medicine, a report from the Bureau of Health (Ministry of Interior) was first published in 1877 and has continued every year since then. In 1872, an official register for each family (including current and official addresses) was prepared and the population for each district and prefecture was published. Later, the Vital Statistics was issued; after then, in 1902 a life table was made public. National census taking — in accordance with an international format — was initiated in 1920. In other words, basic statistical data had been prepared early and statistical publications prepared by statisticians had been in existence. In medicine, although statistics had been used as a methodology, it was recognized as a branch of hygienics and not as a basic course in medical science.

As an episode to illustrate the relationship between medicine and statistics, Shun'ichi Yamamoto introduced the following:

Rintaro Mori, an army physician who had been trained in Germany, understood statistics well and was instrumental in wide application of statistics in the army. In 1889, Shuzo Kure, then a student at Tokyo Imperial University, translated Osterlin's Handbuch der medizinishe Statistik 1874 (Medical Statistics) and asked Rintaro Mori to write a preface for it. Mori in turn summarized the features of statistics and described the usefulness of the book translated by Kure. Takeo Imai, who had studied statistics in areas other than medicine and had been the chief editor of a statistical journal, published his critique stating that the translated word, "statistics" (for Japanese) was neither appropriate nor sufficient to describe the content: he stated that they used the phonetic spelling of statistics in "katakana" because of the lack of a more appropriate expression. Instead of Kure, the translator, Mori, who wrote the preface to the book, responded to this criticism, which started a series of emotional contentions between Mori and Imai. The former stated that statistics was a methodology to show a relationship: it is a probabilistic theory and could not determine a cause-effect relationship. In response to this, Imai rejected this statement by stating that a statistical study, if repeated and arriving at the same result, might be used to estimate a cause-effect relationship. A third party joined in this argument, and it took on the appearance of a mudslinging contest. The argument was carried on by Fumifusa Kure, an older brother of Shuzo Kure and also a statistician. The argument came to an end eventually. Meanwhile, Fumifusa Kure published books on statistics, using the translated word for statistics, which came to be accepted widely. Shun'ichi Yamamoto stated that on the whole, the argument between Mori and Imai had little merit, one of the reasons being that the former was young and self-confident immediately after his return from Germany. Because this argument was about a method to prove the cause-effect relationship, it might have created the misconception that statistics could not explain away such a relationship in medical phenomena. In the academic medical circles of those days, much weight was placed on the results of deterministic analysis: Shun'ichi Yamamoto stated that the argument — such as the one described above — may have put the brakes on studies that conflate medicine and statistics. From the early 20th century, statistics developed rapidly in the western world. In Europe and the United States in the 1930s, its progress had a profound effect on medical research. But a lack of interest in statistics in this country slowed progress in medical research.^53^^-^^54^
